# Determining the Risk Factors of Complications Due to Endoscopic Retrograde Cholangiopancreatography

**DOI:** 10.7759/cureus.51666

**Published:** 2024-01-04

**Authors:** Özhan Özcan, Soykan Arikan

**Affiliations:** 1 General Surgery, Istanbul Education Research Hospital, Istanbul, TUR; 2 Surgical Oncology, Basaksehir Cam and Sakura City Hospital, Istanbul, TUR

**Keywords:** ercp-induced complications, post-ercp bleeding, post-ercp cholecystitis, post-ercp pancreatitis, post-ercp cholangitis, endoscopic retrograde cholangiopancreatography

## Abstract

Background and objective

The effective use of endoscopic retrograde cholangiopancreatography (ERCP) has been on the rise in diagnosing and treating benign and malignant pathologies of the common bile duct and pancreas. ERCP, a complex procedure requiring high knowledge, skills, and practice, differs from other endoscopic applications as it involves the use of different techniques and equipment and the occurrence of more complications. The most commonly observed complications of ERCP are pancreatitis, bleeding, perforation, and infections. In this study, we aimed to assess the incidence of post-ERCP complications to identify the associated risk factors and indications.

Methodology

In this study, 181 ERCP procedures performed on 122 consecutive patients in the endoscopy unit of Istanbul Training Hospital were prospectively evaluated by using an observational method to determine the frequency of and risk factors for post-ERCP complications. The patients were followed up in the course of the ERCP procedure and for 30 days after the procedure; the complications and clinical developments were recorded.

Results

The mean age of the cohort was 59.7 ± 17.7 (19-97) years; 40.9% were female and 59.1% were male. The cannulation success was achieved in 77.3% of the ERCP procedure performed. Among the ERCP procedures applied, 89% were performed for therapeutic purposes, and choledocholithiasis (60.2%) was the most common indication for ERCP. Major complications were detected in 25.4% of the patients after ERCP. The most common major complication was cholangitis (9.9%), followed by pancreatitis (7.2%), cholecystitis (5.5%), bleeding (3.9%), and perforation (1.1%). It was observed that sphincterotomy was associated with an increase in all complications. The incidence of cholangitis decreased in the presence of dilated bile ducts unrelated to obstruction. The increased incidence of pancreatitis was associated with the female gender, the use of sphincterotomy and basket, inexperienced endoscopists, and inpatient admissions. The incidence of cholecystitis, on the other hand, was found to be linked with sphincterotomy and inexperienced endoscopists.

Conclusions

ERCP is a complex endoscopic procedure that requires high technical knowledge and skill and can cause serious complications. For endoscopists to perform clinically effective and accurate ERCP, it is important that they correctly determine the indications for the procedure, know its potential complications, and refrain from practices that will create complications and are unnecessary as much as possible.

## Introduction

Endoscopic retrograde cholangiopancreatography (ERCP) is widely used in the diagnosis and treatment of biliary tract and pancreatic diseases. Its use has gradually increased over time; while its use as a diagnostic method has been abandoned, its therapeutic use has significantly come to the fore [1]. Although biliary pathology treatment modalities have been improved through ERCP, they carry a higher risk of complications compared to other endoscopic procedures. Different ERCP-induced complications with varying incidences have been reported in the literature according to the method, patient selection, and definitions of complications. The incidence of major ERCP-induced complications is as follows: 2-15.1% for pancreatitis, 0.4-10% for cholangitis, 0.1-8.6% for cholecystitis, 0.3-2% for bleeding, 0.5-2.1% for perforation, and around 1% for procedure-related mortality [2-7].

Many prospective and retrospective multicenter studies investigating patient- and procedure-related risk factors in ERCP applications are available in the literature. It is important to fully identify the risk factors for ERCP complications because it enables us to enhance the safety of the procedure and recognize high-risk cases where ERCP should be avoided if necessary [8,9]. This study aims to determine the incidence of post-ERCP complications to identify the risk factors and indications for each complication and to discuss the outcomes of ERCP-related cases included in the study.

## Materials and methods

Patients

This was a single-center, prospective observational study. A total of 122 consecutive patients admitted to our hospital between June 2009 and October 2009 and required diagnostic or therapeutic ERCP procedures were enrolled in the study. Before commencing the study, ethical approval was obtained from the local ethics committee (decision number: 5922/900, dated 23.6.2009 and numbered 28). The patients who would undergo ERCP were informed about the study and their informed consent was obtained.

The inclusion criteria for the study are as follows: outpatients and inpatients who were to undergo ERCP, and whose indications were determined and examined in outpatient clinics and services. Patients whose imaging report (abdominal USG, abdominal MRI and MRCP, abdominal CT) and laboratory results could not be obtained before ERCP, and whose laboratory results and clinical follow-up could not be obtained at the end of the first month after ERCP were excluded from the study.

Study protocol and data collection

The patients' demographic data, risk factors, chronic diseases, prior pancreaticobiliary diseases and interventions, and medications used were recorded on the prepared standard form. Before the ERCP procedure, the radiological and laboratory results of the patients were noted, and the anesthetic risk of the patients before the ERCP procedure was determined according to the criteria of the American Society of Anesthetists (ASA).

All patients were taken to the procedure after fasting for at least six hours per the protocols of the endoscopy unit of our hospital. Prophylactic antibiotics and NSAIDs were not routinely administered. No intervention was made by the endoscopist during ERCP and the interventions of all endoscopists in their own practices were recorded. After vascular access was achieved in all patients, intravenous sedation was performed under midazolam and pethidine for patients who did not undergo general anesthesia; these procedures were not evaluated. Intravenous hyoscine N-butyl bromide was administered as needed to provide duodenal relaxation; blood oxygen saturation and pulse rates were monitored with pulse oximetry throughout the procedure. The medical treatments that the patients underwent were recorded in the patient follow-up forms.

All procedures were performed by a standard method using different papillatomes (Microvasive, Boston, and Wilson-Cook) and pre-cut papillatome when necessary, accompanied by the use of a standard videoendoscope (Fujinon ED-250 XD5 and Pentax ED-3480TK SN A120448). Mechanical lithotripsy, balloon sweeping, and Dormia baskets were employed when necessary to extract the stone. Biopsy forceps and brushes were utilized for biopsy from the papilla and common bile duct. Stents of various diameters and lengths were placed given the suspicion of benign and malignant stenosis and remaining stones. All endoscopic information, complications, and side effects were recorded in the information form throughout the procedure.

After the procedure, all patients were observed in the recovery room for at least 20 minutes. The patients who had no pain, fever, changes in vital signs, and prolonged sedation were discharged. The follow-up of inpatients staying in our hospital was done by visiting them at their sick beds on the first day after ERCP, and the disease course was documented by contacting the attending physician throughout the inpatient stay. The laboratory tests conducted during follow-up and imaging methods were obtained from hospital medical records. For the follow-up of the inpatients in other centers, the patients and their follow-up doctors were contacted by phone, and the information about the disease course was obtained and recorded. The discharged outpatients and their relatives were informed in detail about post-ERCP complications and symptoms; those who had these symptoms were followed up as inpatients, and their results were recorded in the patient follow-up form. For unproblematic ERCP cases, the patients were contacted at the end of day one, day seven, and day 30, and their conditions were recorded. Follow-up periods and duration were increased for those who required repeat ERCP and developed complications.

Observation variables and definitions of complications

The variables considered to be associated with the risk of complications after ERCP were classified into four groups in the patient follow-up form: patient-related factors, disease- or structure-related factors, application-related factors, and the endoscopist’s experience.

In our study, the definition of diagnostic ERCP included cannulation and injection of contrast material, as well as biopsy or brush cytology to identify the biliary and pancreatic ductal systems, called cholangiopancreatography. The definition of therapeutic ERCP included procedures such as endoscopic sphincterotomy, precutting, drainage, stenosis dilation, stent placement, oddi sphincter manometer measurement, and gallstone removal, either alone or in combination.

Schmit et al.'s [10] classification was used to evaluate the success of ERCP. Complete success was defined as success in fully meeting the criteria for indications and diagnoses for treatment. Partial success was defined as the failure to meet the criteria for some diagnostic or treatment indications during the procedure, regardless of whether the patient was referred for a second ERCP. Complete failure was defined as the failure to meet the criteria for any diagnostic or treatment indications.

Hospitalization for any undesirable cause that was clearly related to ERCP, as defined by Vandervoort et al., was deemed a complication [9]. Each of the ERCP-related complications evaluated in our study was defined. Pancreatitis was defined as new-onset abdominal pain and a fourfold increase in serum amylase or lipase within 24 hours of the procedure. Bleeding was defined as the presence of clinical signs of bleeding along with the need for papillotomy or endoscopic and other homeostasis methods due to a decrease in hemoglobin value of more than 2 g/dL. Cholangitis was defined as fever and chills with increased liver enzymes and/or positive blood culture within 24 hours after the procedure. Cholecystitis was defined as inflammation of the gallbladder detected clinically and radiologically. Perforation was defined as a condition where free or retroperitoneal air was detected radiologically. Hyperamylasemia was defined as serum amylase value exceeding the normal level, not suggestive of any symptoms of pancreatitis.

The severity levels of complications were classified as follows: mild complications (requiring one to three days of hospitalization), moderate complications (requiring 4-10 days of hospitalization), and serious complications (requiring more than 10 days of hospital stay, surgical or radiological intervention, or those causing death), according to the classification devised by Freeman et al., based on the duration of hospital stay and the severity of the required intervention.

Endoscopists were divided into two groups, as per the literature, according to ERCP-related experience: those who performed sphincterotomy twice or more per week on average were considered experienced, and those who performed sphincterotomy once a week or less were regarded as inexperienced [2].

Statistical analysis

SPSS Statistics for Windows 16.0 software (IBM Corp., Armonk, NY) was employed for the statistical analyses of the results obtained in the study. In evaluating the study data, along with descriptive statistical methods (mean, standard deviation, frequency, percentage distribution), the Student's t-test and Chi-square test were used in the analysis of the quantitative and qualitative data, respectively. The studied variables comprised age, gender, indication for ERCP, admission type, the purpose of the procedure, endoscopist experience, ERCP duration and cannulation time, first/repeat ERCP, ERCP procedures (sphincterotomy, balloon, basket, Wirsung cannulation, Wirsung contrast, pre-cut, stent, stone extraction). Logistic regression analysis was employed for the determination of the risk factors considered to have an effect on the dependent variable. The statistical tests were two-sided and a p-value <0.05 was regarded as statistically significant.

## Results

A total of 181 ERCP procedures were performed on 122 patients, including one ERCP for 83 patients, two ERCPs for 27 patients, three ERCPs for eight patients, four ERCPs for three patients, and eight ERCPs for one patient. Of these, 100 (55.2%) were performed on outpatients and 81 (44.8%) on inpatients. Among the patients, 40.9% were female and 59.1% were male, and the overall mean age was found to be 59.7 ± 17.7 (19-97) years; 57.4% of the patients were classified as ASA-1, 31.1% as ASA-2, 9.0% as ASA-3, and 2.5% as ASA-4. At least one imaging report of each patient was accessed before ERCP, and 170 abdominal USG, 108 upper abdominal MRI/MRCP, and 43 abdominal CT information were recorded on the patient follow-up forms. The outcomes of ERCP procedures performed were as follows: complete success in 124 (68.5%) cases, partial success in 12 (6.6%) cases, and failure in 45 (24.8%) cases.

Successful ERCP cannulation was performed in 140 (77.3%) procedures by using the standard cannula in 92 procedures, and standard sphincterotomy in 42 patients, while both were used in six patients. There were 98 (54.1%) first-time ERCP procedures and 83 (47.9%) repeat procedures in the series. ERCP was performed for diagnostic purposes in 20 (11%) cases and for therapeutic purposes in 161 (88.9%) cases. Experienced endoscopists were involved in 119 (65.7%) cases and inexperienced endoscopists in 62 (34.3%) cases. The mean ERCP time was found to be 22.6 ± 12.0 minutes; 36 cases were completed in ≥30 minutes and 145 cases in <30 minutes. The mean cannulation time was 17.7 ± 10.8 minutes; it was completed in ≥30 minutes in 21 cases and <30 minutes in 160 cases (Table 1).

**Table 1:** Demographics of the patients and their ERCP-related information ERCP: endoscopic retrograde cholangiopancreatography

Demographics and ERCP information		N (%)
Gender	Male	65 (59.1)
	Female	57 (40.9)
Admission type	Outpatient	100 (55.2)
	Inpatient	81 (44.8)
Procedure purpose	Diagnostic	20 (11.1)
	Therapeutic	161 (88.9)
ERCP success	Complete success	124 (68.5)
	Partial success	12 (6.6)
	Failure	45 (24.8)
Endoscopist experience	Experienced	119 (67.5)
	Inexperienced	62 (34.3)
ERCP duration, minutes	<30	145 (80.2)
	≥30	36 (19.8)
Cannulation time, minutes	<30	160 (88.4)
	≥30	21 (11.6)
First/repeat ERCP	First	98 (54.1)
	Repeat	83 (45.9)

The indications for ERCP procedure at admission were as follows: choledocholithiasis (n=109, 60.2%), malignancy (n=24, 13.3%), pancreatitis (n=18, 9.9%), biliary fistulas (n=17, 9.4%), and other causes (n=18, 9.9%), respectively (Table 2). The most commonly administered therapeutic intervention during ERCP was a ballon sweep of the common biliary duct; other therapeutic interventions are presented in Table 2. Among the 23 cases with biliary stent placement, 10 had malignant stenosis, 13 had residual stones in the common bile duct, and stent revision was performed in three patients.

**Table 2:** Therapeutic techniques used in ERCP ERCP: endoscopic retrograde cholangiopancreatography

Techniques used in ERCP	N (%)
Balloon use	110 (60.8)
Standard biliary sphincterotomy	86 (47.5)
Basket use	84 (46.4)
Biliary stent placement and revision	23 (12.7)
Use of pre-cut sphincterotomy	15 (8.3)
Use of mechanical lithotripter	12 (6.6)
Biopsy extraction	9 (4.9)

Major complications were detected in 46 (25.4%) of 181 ERCP procedures. The total number of complications was 50 (27.6%) (more than one complication was seen in some cases). The distribution of complications depending on the degree of severity in terms of mild/moderate/severe categorization was as follows: cholangitis was detected in 18 (9.9%) (15/3/0) patients, pancreatitis in 13 (7.2%) (9/3/1) patients, cholecystitis in 10 (8/2/0) (5.5%), bleeding in seven (3.9%) (6/1/0) patients, and perforation in two (1.1%) (0/0/2) patients. All complications were treated conservatively except for two cases where perforation was detected.

A univariate analysis of the development of complications depending on the variables was performed, and patient-related factors, disease/structure-related factors, endoscopist’s experience, and ERCP procedure types are shown in Tables 3-4. It was determined that the development of cholangitis significantly decreased in patients with a diagnosis of dilated common bile duct of unknown origin (p=0.05). The increase in pancreatitis development was found to be statistically significantly associated with female gender (p=0.007), sphincterotomy procedure (p=0.026), basket use (p=0.023), inexperienced endoscopists (p=0.05), and interventions applied to inpatients (p<0.001). It was determined that the increase in the development of cholecystitis was statistically significantly associated with the sphincterotomy procedure (p=0.030) and inexperienced endoscopists (p<0.001). The evaluation of ERCP-induced bleeding and perforation indicated no statistical significance between the variables. The total complications after ERCP increased significantly with sphincterotomy performed (p<0.001), and it was determined that the effect of endoscopist experience in the development of complications was not significant in the experienced endoscopists, while it was close to significant in the inexperienced endoscopists (p=0.059) (Tables 3, 4).

**Table 3:** Distribution of ERCP-induced complications based on the variables ERCP: endoscopic retrograde cholangiopancreatography

		Cholangitis, n=18, n (%)/p-value		Pancreatitis, n=13, n (%)/p-value		Cholecystitis, n=10, n (%)/p-value		Bleeding, n=7, n (%)	Perforation, n=2, n (%)
Female		7 (38.8)		10 (76.9)	p=0.007	5 (50.0)		3 (42.8)	2 (100)
Male		11 (61.1)		3 (23.0)		5 (50.0)		4 (57.1)	0
Experienced endoscopist		13 (72.2)		4 (30.7)		1 (10.0)		5 (71.4)	0
Inexperienced endoscopist		5 (27.7)		9 (69.2)	p=0.005	9 (90.0)	p <0.001	2 (28.5)	2 (100)
Diagnostic ERCP		1 (5.5)		1 (7.6)		0		2 (28.5)	0
Therapeutic ERCP		17 (94.4)		12 (92.3)		10(100)		5 (57.1)	2 (100)
Inpatients		8 (44.4)		12 (92.3)	p<0.001	4 (40.0)		3 (42.8)	2 (100)
Outpatients		10 (55.5)		1 (7.6)		6 (60.0)		4 (57.1)	1 (50.0)
ERCP indications	Normal	3 (16.6)		4 (30.7)		1 (7.6)		1 (14.2)	0
	Choledocholitiais	6 (33.3)		6 (46.1)		1 (5.2)		1 (14.2)	1 (50.0)
	Malignancy	3 (16.6)		1 (7.6)		0		0	0
	Dilated bile duct	1 (5.5)	p=0.05	4 (30.7)		3 (30.0)		3 (42.8)	1 (50.0)

**Table 4:** Evaluation of ERCP-induced complications based on ERCP procedure type ERCP: endoscopic retrograde cholangiopancreatography

ERCP procedure	Cholangitis, n=18, n (%)	Pancreatitis, n=13, n (%)/p-value		Cholecystitis, n=10, n (%)/p-value		Bleeding, n=7, n (%)	Perforation, n=2, n (%)	Total, n=50, n (%)/p-value	
Sphincterotomy	10 (55.5)	11 (84.6)	p=0.026	9 (90.0)	p=0.030	5 (71.4)	1 (50.0)	36 (72.0)	p<0.001
Balloon	8 (44.4)	11 (84.6)		9 (90.0)		6 (85.7)	1 (50.0)	35 (70.0)	
Basket	6 (33.3)	10 (76.9)	p=0.023	7 (70.0)		4 (57.1)	2 (100)	29 (58.0)	
Wirsung cannulation	2 (11.1)	4 (30.7)		2 (20.0)		1 (14.2)	0	9 (18.0)	
Wirsung contrast	1 (0.55)	3 (23.0)		1 (10.0)		1 (14.2)	0	6 (12.0)	
Pre-cut	1 (0.55)	0		0		1 (14.2)	0	2 (4.0)	
Stent	5 (27.7)	2 (15.3)		1 (10.0)		0	0	8 (16.0)	
Stone extraction	6 (33.3)	6 (46.1)		4 (40.0)		1 (14.2)	1 (50.0)	18 (36.0)	

Also, multivariate logistic regression analysis was performed involving the parameters considered to be associated with all ERCP-induced complications. The increase in the development of pancreatitis was statistically significantly associated with female gender, practices of inexperienced endoscopists, basket application, and inpatient admissions. It was detected that the development of cholecystitis was statistically significantly higher in the patients whose ERCP procedures were performed by inexperienced endoscopists. On the other hand, no significance was found in the multivariate analysis of other complications with variables.

In the t-test, in which the independent groups were evaluated with quantitative parameters for ERCP-induced complications, it was observed that patient age, cannulation time, ERCP time, amount of contrast applied, length of sphincterotomy performed, and balloon inflation amount closely distributed for each and total complications, and those parameters did not create a significant difference. In terms of the total complications after ERCP, no significant difference was observed in the multivariate logistic regression analysis, which included gender, endoscopist experience, ERCP time, balloon inflation amount, and sphincterotomy parameters.

Papillary bleeding requiring endoscopic intervention was observed in seven (3.9%) cases during ERCP. Of these, bleeding was stopped using sclerotherapy in three, coagulation cautery in two, balloon compression in one, and both coagulation and sclerotherapy in one. One patient was followed up by hospitalization, but no transfusion was required.

ERCP-induced perforation was detected in two patients. One of them was a 90-year-old female patient whose stone from the distal common bile duct was extracted using sphincterotomy, basket, and balloon application; a biliary stent was tried, but the procedure failed. During the procedure, perforation was observed at the esophagogastric junction. Emergency laparotomy was performed on the patient; a 5-cm perforation was detected at the cardioesophageal junction, and it was repaired primarily. Pancreatitis developed during the ICU follow-up, and the patient died on the fourth day post-procedure. The other patient with perforation was a 65-year-old cholecystectomized female patient who had previously undergone ERCP due to choledocholithiasis, and a stent had been placed because of residual stone. ERCP was attempted for stent replacement in the patient; the old stent was removed but the new stent could not be placed; air was detected in the retroperitoneum in the abdominal CT taken due to the suspicion of stent-induced perforation in duodenum. The perforation site of the patient could not be detected in the laparotomy performed; the remaining common bile duct stones were extracted by common bile duct exploration, and the patient was discharged on the fifth postoperative day without any issues.

The minor complications seen during ERCP were as follows: hypoxia in two patients, arrhythmia in two patients, and anxiety in three patients. ERCP could not be completed in two patients who developed anxiety; other minor complications resolved during ERCP. Hyperamylasemia was detected in 37 (20.4%) cases, and their follow-up was non-problematic. A total of six patients died during the follow-up period; ERCP-related mortality occurred in two (1.6%) patients, one with perforation and pancreatitis, and the other with pancreatitis.

## Discussion

ERCP is essentially a therapeutic procedure with many indications, and it is usually employed in pancreaticobiliary disorders; it is considered to be relatively complicated due to the requirement of specialized equipment and the fact that it takes a long time to develop proficiency in it [11]. Despite the benefits it provides as a minimally invasive approach in the treatment of bile and pancreatic disorders, it is associated with the highest risk among endoscopic procedures, with a higher potential for complications than other standard endoscopic interventions [12]. In our study, major complications were detected in 46 (25.4%) of 181 ERCP procedures. The total number of complications was 50 (27.6%) since more than one complication was seen in a few patients. It has been reported in the literature that the overall incidence of ERCP-related complications ranges from 5 to 20.5% [2,9,13,14]. The wide range in the reported incidences may be attributed to the heterogeneity of the definition and inclusion criteria of ERCP-related complications, the inclusion of diagnostic ERCP, and the difference in endoscopists' experience.

In most of the studies on post-ERCP complications, the data collection has focused on early complications, and the detection of late post-procedure complications has generally been overlooked. In addition, more information is obtained and a higher number of complications are identified in prospective studies compared to retrospective studies and in single-center studies compared to multicenter studies [15]. In our study, the long follow-up period and single-center design enabled us to detect some mild complications such as cholangitis and cholecystitis cases that occur due to increased risk of ascending infection in the late period. In the definitions of complications in many publications, ERCP-induced papillary bleeding was not considered a complication unless it caused clinical symptoms [6]; in our study, on the other hand, bleeding that occurred in connection with sphincterotomy and pre-cut and required endoscopic intervention was regarded as a complication. The fact that complication rates in our series are higher than those reported in the literature may be due to the long follow-up period, differences in the definition of complications, and the single-center study design.

The incidence of post-ERCP cholangitis varies from 0.4% to 10% in the literature according to the population studied [5]. One of the mechanisms of this infection is defined as bile colonized with bacteria or endotoxins, called biliary stasis, crossing the blood barrier during prolonged ERCP procedures. Another mechanism involves the damage of the epithelium during contrast injection or other procedures that allow bacteria to enter [2,16]. The development of cholangitis is more commonly observed in the presence of difficult and insufficiently drained bile ducts due to malignant hilar occlusion [17]. The most commonly identified complication in our study was the development of cholangitis, and it was detected in 18 (9.9%) patients: 15 of which were mild and three were moderate in severity. Although cholangitis was seen more frequently in patients with malignancy and stent placement, this was determined to be statistically insignificant. Our results are generally consistent with the findings in the literature, but the incidence of cholangitis exceeded the average in the literature. We believe that the long follow-up period enabled us to detect cholangitis cases that occur due to the increased risk of ascending infection in the late period.

Pancreatitis is the most common complication of ERCP. The original consensus definition and classification of post-ERCP pancreatitis was first reported by Cotton et al. [6] in 1991. The second definition entails the revised Atlanta International Consensus (RAC) definition and the acute pancreatitis (AP) classification. Its incidence varies between 2% and 15.1% in prospective series where the common consensus definition is used [7,18]. In our study, pancreatitis (7.2%) was the second most common post-ERCP complication, and it was detected in 13 patients, classified as mild in nine, moderate in three, and severe in one. In the literature, many mechanisms have been suggested for pancreatitis, including direct mechanical trauma due to prolonged or difficult instrument manipulation causing ductal edema, chemical injury due to injected contrast, and hydrostatic injury due to increased pressure in the pancreatic duct due to manometry. Intestinal flora as a source of infection may represent a mechanism of injury other than the endoscope or contrast agent in the pancreatic duct. In addition, pancreatitis may also be a thermal injury occurring due to the use of electrocautery during sphincterotomy [19].

The operator-, patient- and method-related independent factors for post-ERCP pancreatitis have been assessed in many studies [20]. Endoscopists with low case volumes are considered to be the main operator-related factor. The patient-related factors include young age, female gender, normal serum bilirubin level, current pancreatitis, history of post-ERCP pancreatitis, absence of chronic pancreatitis, sphincter of Oddi disorder, small bile duct, dilated bile duct, pancreatic divisum, and history of laparoscopic cholecystectomy. The reported method-related factors are difficult cannulation, administration of contrast to the pancreatic duct, number of pancreatic contrast injections, performing sphincter of Oddi manometry, performing pre-cut sphincterotomy, inability to clear stones from the common bile duct, use of sphincterotomy, inadequate sphincterotomy, pain during the procedure, stent placement, brush cytology, and biliary sphincteroplasty [20,21]. In our study, consistent with the literature, the increase in the development of pancreatitis was statistically significantly associated with female gender and practices of inexperienced endoscopists. We consider that the thermal damage caused by sphincterotomy, and papillary edema developed due to mechanical trauma created by manipulation in basket applications increase the risk of pancreatitis. However, univariate and multivariate analyses conducted as part of our study indicated that the effects of young age, history of cholecystectomy and pancreatitis, wirsung cannulation, and wirsung contrast imaging were found to be insignificant on the development of pancreatitis.

Many publications have reported that the risk of pancreatitis in therapeutic ERCP is higher than that performed for diagnostic purposes, and its incidence is approximately two times higher [22]. In our study, there was no statistically significant difference in terms of the development of pancreatitis between diagnostic and therapeutic ERCPs, which may be attributed to the fact that there was a lower number of cases with diagnostic ERCP.

In the prevention of post-ERCP pancreatitis, the use of prophylactic pancreatic duct stenting, especially in high-risk patients, has been shown to increase the efficiency of guidewire-assisted cannulation, restrict contrast injection into the pancreas, reduce the need to use pre-cut sphincterotomy, and limit other ERCP-related complications [23,24]. Furuya et al. showed that papillary fistulotomy is more effective than guidewire cannulation in maintaining low amylase/lipase levels to overcome complex bile duct cannulation [25]. Various medical treatments have been suggested for preventing post-ERCP pancreatitis. NSAIDs inhibit the key mediators (prostaglandins and phospholipase A2) involved in the development of post-ERCP pancreatitis. A meta-analysis of 21 randomized controlled trials indicated that the administration of indomethacin after ERCP is more effective in preventing pancreatitis compared to its administration before ERCP [26]. In our study, routine NSAID application was not observed and was not evaluated.

Post-ERCP cholecystitis was detected in 10 patients (5.5%), and two of them were classified as moderately severe. Studies have reported that the incidence of acute cholecystitis varies between 0.1% and 8.6% [2-4]. Its pathogenesis may depend on the administration of non-sterile contrast material to the poorly emptied gallbladder and mechanical or inflammatory obstruction of the bile duct due to endoprosthesis, stone, and malignancy [3,27]. In high-risk patients, temporary gallbladder decompression with the use of a plastic stent or endoscopically placed nasocolecystic tube should be considered. The benefit of prophylactic antibiotic administration in preventing cholecystitis has yet to be demonstrated [21]. In our study, it was determined in the univariate analysis in which the patient, endoscopist, disease, and risk factors of the procedure were evaluated that the incidence of increase in post-ERCP cholecystitis was only significantly associated with the practices of inexperienced endoscopists and the use of sphincterotomy. On the other hand, the variables considered as possible risk factors, namely the patient's history of cholecystitis and cholangitis before ERCP, the use of stenting, and the presence of malignancy, were not statistically significantly associated with an increase in the development of cholecystitis after ERCP. In addition, the use of prophylactic antibiotics did not statistically reduce the incidence of post-ERCP cholecystitis.

The most commonly reported causes of ERCP bleeding are endoscopic biliary and pancreatic sphincterotomy. The bleeding rate after sphincterotomy is 0.3-2% [3,6,21]. In the prospective multicenter series carried out by Freeman et al. [2], bleeding was observed in 48 of 2347 ERCP patients (2%) who underwent biliary endoscopic sphincterotomy. Of these cases, 14 were mild, 22 were moderate, and 12 were severe, whereas mortality occurred in two patients with Child-Pugh class C cirrhosis. In patients who underwent sphincterotomy for ERCP, anticoagulant intake, platelet count of less than 50,000/mm^3^, cirrhosis, use of dialysis for end-stage renal disease, bleeding during the procedure, and practices of less experienced endoscopists have been deemed risk factors [21]. In our study, ERCP was not applied to those with INR (international normalized ratio) prolongation, which is considered to be a risk factor for bleeding, and those who took aspirin and drugs that could cause coagulation disorders within the last three days. As we regarded all bleedings requiring endoscopic intervention as complications, independent of clinical signs, the bleeding incidence we found was higher than the results reported in the literature in recent years. Sphincterotomy was performed in 71.4% of the patients with bleeding, but it did not have any statistical significance.

In various large series, the incidence of perforation has been detected to be 0.5-2.1% in ERCP patients [6,9,28]. Four types of perforation are described in the Stapfer classification, a scheme used for classifying ERCP-related perforations: canal or tumor perforation caused by guide wire and other instruments, also called penetration; sphincterotomy-related retroduedonal perforation; endoscopic perforation in the esophagus, stomach, or duodenum (away from the papilla); and stent-related perforations. Among those, retroperitoneal duodenal perforation is the most frequently seen variant [29]. The incidence of perforation has been recently reported to be below 0.5%, thanks to the increasing experience and skills of endoscopists. Nevertheless, severe and fatal complications are still observed to occur. There were two (1.1%) patients with ERCP-related perforation in our series, namely endoscopy-related distal esophageal perforation, and stent-related retroduodenal perforation. The patient with distal esophageal perforation died due to pancreatitis and sepsis after an emergency laparotomy. In our other patient, the stone had been extracted from the common bile duct with the use of sphincterotomy, basket, and balloon exploration, and a stent had been placed due to the remaining stone in the previous ERCP. ERCP was tried for stent replacement of the patient; while the old stent was removed, the new one could not be placed. The presence of air was detected in the retroperitoneum in the abdominal CT taken due to suspicion of stent-related perforation in the duodenum. The laparotomy performed on the patient could not establish the location of the perforation, and the remaining common bile duct stones were extracted with common bile duct exploration and T-tube application. The patient was eventually discharged on the fifth postoperative day without any problems. Since post-ERCP abdominal CT was not performed for asymptomatic patients in our study, we think that possible asymptomatic perforations due to sphincterotomy may have been overlooked.

It was observed that sphincterotomy performed during ERCP significantly increased the total number of complications. The practices of inexperienced endoscopists in our study created a significant increase in the development of pancreatitis and cholecystitis and also led to an increase that approached significance in the total number of complications. The absence of any significant effect of the practice of experienced endoscopists on all complications could be attributed to the fact that the majority of cases (67.5%), as well as ERCP cases that had been tried and failed in other centers, besides the complicated ones were performed by experienced endoscopists at our hospital. Difficult cannulation and technical challenges are cited as risk factors for all complications [5,30]. In our study, ERCP time and cannulation time were assessed to be independent risk factors, which reflects the difficulty of the procedure and the need for applied technical maneuvers. In the univariate analysis, it was observed that the ERCP time, cannulation time as well as the difference between ERCP and cannulation times increased in cases with developing complications; however, this increase was not statistically significant.

There is a risk that endoscopy-related bacteremia can spread to distant tissues and organs. Among the most common infections following ERCP are cholangitis, cholecystitis, duodenoscope-related infection, and, to a lesser extent, infective endocarditis. Bacteria enter the common bile duct via the hematogenous route or more often by reflux. Anatomical barriers in patients with normal bile ducts block both routes. In addition, immunocompromised patients and those with impaired bacterial barriers due to biliary obstruction are much more susceptible to these complications. Biliary sphincterotomy is an important step in therapeutic ERCP procedures; however, it disrupts the anatomy and defense mechanism of the common bile duct, thereby making the common bile duct and gallbladder more susceptible to pathogens in the intestinal flora. The incidence of post-ERCP infective complications in our study was higher than that in the literature, and this can be explained by the prospective and single-center nature of our study. The follow-up and treatments of these patients' complaints suggestive of ERCP-induced infection were evaluated, and this enabled us to document many late infective complications.

## Conclusions

Although ERCP is the standard treatment method for various disorders of the pancreaticobiliary system, it is also associated with potentially life-threatening serious complications. In our study, the risk factors that affect ERCP-induced complications were as follows: female gender, the use of sphincterotomy and basket, practices by inexperienced endoscopists, and inpatient admissions. Additionally, the higher complication rate observed compared to the literature may be due to the long follow-up period, differences in complication definitions, and single-center study design. For endoscopists to be able to perform clinically effective and accurate ERCP, it is important that they correctly determine the indications for the procedure, know its potential complications, and avoid as much as possible the practices that will create complications and are unnecessary. It is imperative to take the necessary precautions for a complication that could occur during the procedure or develop post-ERCP and follow up with patients after the procedure, especially to detect infective complications.
